# Acute Kidney Injury Predicts Mortality after Charcoal Burning Suicide

**DOI:** 10.1038/srep29656

**Published:** 2016-07-19

**Authors:** Yu-Chin Chen, Yi-Chia Tseng, Wen-Hung Huang, Ching-Wei Hsu, Cheng-Hao Weng, Shou-Hsuan Liu, Huang-Yu Yang, Kuan-Hsin Chen, Hui-Ling Chen, Jen-Fen Fu, Wey-Ran Lin, I-Kuan Wang, Tzung-Hai Yen

**Affiliations:** 1 Center for Traditional Chinese Medicine, Chang Gung Memorial Hospital, Taoyuan, Taiwan; 2 Department of Nephrology and Division of Clinical Toxicology, Chang Gung Memorial Hospital and College of Medicine, Chang Gung University, Linkou, Taiwan; 3 Kidney Research Center, Chang Gung Memorial Hospital, Linkou, Taiwan; 4 Department of Dentistry and Craniofacial Orthodontics, Chang Gung Memorial Hospital, Linkou, Taiwan; 5 Department of Medical Research, Chang Gung Memorial Hospital and College of Medicine, Chang Gung University, Linkou, Taiwan; 6 Department of Gastroenterology, Chang Gung Memorial Hospital and College of Medicine, Chang Gung University, Linkou, Taiwan; 7 Department of Nephrology, China Medical University Hospital and College of Medicine, China Medical University, Taichung, Taiwan; 8 Center for Tissue Engineering, Chang Gung Memorial Hospital, Linkou, Taiwan

## Abstract

A paucity of literature exists on risk factors for mortality in charcoal burning suicide. In this observational study, we analyzed the data of 126 patients with charcoal burning suicide that seen between 2002 and 2013. Patients were grouped according to status of renal damage as acute kidney injury (N = 49) or non-acute kidney injury (N = 77). It was found that patients with acute kidney injury suffered severer complications such as respiratory failure (P = 0.002), myocardial injury (P = 0.049), hepatic injury (P < 0.001), rhabdomyolysis (P = 0.045) and out-of-hospital cardiac arrest (P = 0.028) than patients without acute kidney injury. Moreover, patients with acute kidney injury suffered longer hospitalization duration (16.9 ± 18.3 versus 10.7 ± 10.9, P = 0.002) and had higher mortality rate (8.2% versus 0%, P = 0.011) than patients without injury. In a multivariate Cox regression model, it was demonstrated that serum creatinine level (P = 0.019) and heart rate (P = 0.022) were significant risk factors for mortality. Finally, Kaplan-Meier analysis revealed that patients with acute kidney injury suffered lower cumulative survival than without injury (P = 0.016). In summary, the overall mortality rate of charcoal burning suicide population was 3.2%, and acute kidney injury was a powerful predictor of mortality. Further studies are warranted.

Charcoal burning suicide was firstly introduced by media to the crowd as a painless and peaceful suicide method in 1998, and soon overwhelmed several Asia countries^1^. In the East/Southeast areas, charcoal-burning suicide significantly increased in Taiwan, Hong Kong, Japan, Korea, and Singapore, but not in Malaysia, Philippines and Thailand. Media reports of charcoal-burning suicide played a role in spreading the idea, and there was an increase of charcoal-burning suicide incidence^2^,^3^. It was proposed that widespread media coverage of this apparent painless suicide method and easy access to barbecue charcoal may have contributed to the epidemic in Asia countries^3^. The rise of charcoal-burning suicide in Taiwan started at 2000^1^, and the method soon occupied 10 out of 22 cities/counties in 2006 as the leading suicide method^2^. The increase of charcoal-burning suicide influenced mainly in urban areas than rural areas, and metropolitan regions had the highest rate^3^. In addition, Taiwan showed the largest magnitude of increase during 1995/1996–2011, about 65-fold increase in rate in affected Asian countries^1^. That made an increase of 39% suicide rates during 1998–2002 in urban Taiwan^4^. Moreover, suicide by gases (mainly charcoal-burning) also showed a marked increase in suicide mortality data recently, which caused 6,822 deaths during the period form 1999–2007 in Taiwan^3^, and had become a severe public health problem^5^.

Acute kidney injury is a common and serious complication that is associated with high mortality rates in critically ill patients. The primary causes of acute kidney injury include ischemia, hypoxia, or nephrotoxicity^6^. Carbon monoxide toxicity is the result of a combination of tissue hypoxia-ischemia secondary to carboxyhemoglobin formation and direct carbon monoxide-mediated damage at a cellular level^7^. The carbon monoxide is a colorless, odorless, nonirritant gas that accounts for numerous cases of carbon monoxide poisoning every year from a variety of sources of incomplete combustion of hydrocarbons such as indoor burning of charcoal burning briquettes^8^. An underlying feature of acute kidney injury is a rapid decline in glomerular filtration rate usually associated with decreases in renal blood flow. Inflammation represents an important additional component of acute kidney injury leading to the extension phase of renal injury^6^. The kidney shows a remarkable discrepancy between blood supply and oxygenation^9^. Despite high blood flow and oxygen delivery, oxygen tensions in the kidney are comparatively low, in particular in the renal medulla. The reason for this lies in the parallel arrangement of arterial and venous pre-glomerular and post-glomerular vessels, which allow oxygen to pass from arterioles into the postcapillary venous system via shunt diffusion. The limitation in renal tissue oxygen supply renders the kidney susceptible to hypoxia and has long been recognized as an important factor in the pathogenesis of acute kidney injury^9^.

There is a paucity of literature exists on risk factors for mortality in charcoal burning suicide, although there were many valuable studies that analyzing the risk factors for mortality in carbon monoxide (CO) poisoning. Charcoal burning was an important portion of suicide mortality and suicide rates for decades, and the scale of spreading and increasing of this method was also huge. One study reported that arterial pH and intubation were related to short-term mortality in CO poisoning^10^, and another study reported myocardial injury was significantly related to long-term mortality in CO poisoning^11^. In addition, our recent study^12^ also revealed that shock status was a predictor of mortality in Taiwanese patients with CO poisoning. The non-survivors suffered greater incidences of hypothermia (P < 0.001), respiratory failure (P < 0.001), shock (P < 0.001), hepatitis (P = 0.016), renal failure (P = 0.003) and coma (P < 0.001) than survivors^12^. Thus, we hypothesized that there could be some physiological biomarkers such as acute kidney injury that may be associated with mortality after charcoal burning suicide.

Therefore, the objective of this study was to examine the clinical features, physiological markers, and clinical outcomes after charcoal burning suicide and the associations between these findings. Most importantly, we sought to assess the association between acute kidney injury and mortality in patients after charcoal burning suicide.

## Results

Table 1 describes the baseline characteristics of 126 patients with charcoal burning suicide, stratified according to status of renal damage as acute kidney injury (creatinine 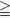
1.2 mg/dL, N = 49) or non-acute kidney injury (creatinine < 1.2 mg/dL, N = 77) group. The patients aged 36.8 ± 11.7 years and 66.7% were male. The majorities of patients with acute kidney injury were male (81.6% versus 57.1%, P = 0.004) and had personal hobbies of smoking (59.2% versus 41.6%, P = 0.019) and alcohol consumption (49.0% versus 40.3%, P = 0.04) than patients without acute kidney injury. Otherwise, there were no significant differences in other baseline variables (P > 0.05).

It was revealed that 46.0% of the charcoal burning suicide patients was single, 11.9% were divorced, 11.9% living alone, 42.1% senior high school educated, 31.7% were jobless, 21.4% had previous suicide history, 49.2% had depressive disorder, 39.7% had adjustment disorder, and 11.1% had substance abuse disorder (Table 2). In addition, there were more single (53.1% versus 41.6% P = 0.009) and divorced (20.4% versus 6.5%, P = 0.009) in the patients with acute kidney injury than without acute kidney injury.

As shown in Table 3, patients with acute kidney injury suffered from severer medical complications, i.e., fever (40.8% versus 19.5%, P = 0.009), acute respiratory failure (49.0% versus 22.1%, P = 0.002), acute myocardial injury (42.9% versus 26.0%, P = 0.049), acute hepatic injury (55.1% versus 22.1%, P < 0.001), acute rhabdomyolysis (49.0% versus 31.2%, P = 0.045) and out-of-hospital cardiac arrest (6.1% versus 0%, P = 0.028) than patients without acute kidney injury.

Patients with acute kidney injury demonstrated a higher heart rate than patients without acute kidney injury (102.2 ± 27.3 versus 92.0 ± 23.3, P = 0.030, Table 4). Additionally, patients with acute kidney injury had poorer laboratory data, i.e., white blood count (19847.1 ± 9180.8/mm^3^ versus 15112.0 ± 12834.5/mm^3^, P = 0.027), blood urea nitrogen (31.2 ± 33.7 mg/dL versus 11.3 ± 2.8 mg/dL, P < 0.001), creatinine (2.7 ± 2.4 mg/dL versus 0.9 ± 0.2 mg/dL, P < 0.001), creatinine kinase (63010.5 ± 11317.0 ng/mL versus 8664.7 ± 1291.7, P = 0.011), myoglobin (24012.9 ± 59481.9 ng/mL versus 1305.3 ± 5343.1, P = 0.022), troponin I (5.4 ± 7.5 versus 1.8 ± 4.9, P = 0.003), aspartate aminotransferase (404.5 ± 966.5 U/L versus 72.2 ± 93.1 U/L, P = 0.005), alanine aminotransferase (200.4 ± 400.5 U/L versus 49.0 ± 44.4 U/L, P = 0.016) and total bilirubin (1.2 ± 1.0 mg/dL versus 0.5 ± 0.2 mg/dL, P = 0.030) than patients without acute kidney injury. Moreover, arterial blood gas analysis also showed a higher degree of metabolic acidosis in the patients with acute kidney injury than without acute kidney injury (pH 7.3 ± 0.1 versus 7.4 ± 0.6, P < 0.001; HCO_3_ 18.6 ± 5.1 versus 21.8 ± 3.5, P P < 0.001).

The overall mortality rate in our charcoal burning suicide population was 3.2% (Table 5). It was demonstrated that patients with acute kidney injury received less hyperbaric oxygen therapy (26.5% versus 50.6%, P = 0.007), but suffered longer duration of hospitalization (16.9 ± 18.3 versus 10.7 ± 10.9, P = 0.002) and higher mortality rate (8.2% versus 0%, P = 0.011) than patients without acute kidney injury.

**Figure 1:**
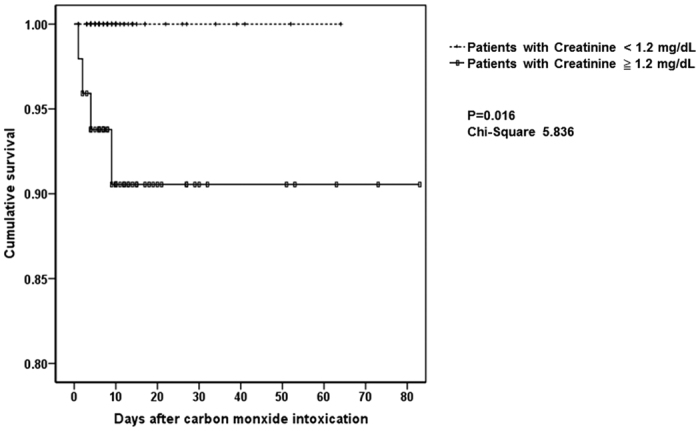
Kaplan-Meier survival analysis. The analysis revealed that patients with acute kidney injury (creatinine 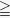 1.2 mg/dL) suffered lower cumulative survival than patients without acute kidney injury (creatinine < 1.2 mg/dL), log-rank test, Chisquare = 5.836, P = 0.016.

In a Cox regression model Table 6, it was disclosed that serum creatinine level (odds ratio 1.761, confidence interval 1.097–2.828, P = 0.019) and heart rate (odds ratio 1.117, confidence interval 1.016–1.228, P = 0.022) were significant risk factors for mortality. In other word, each increment of 1.0 mg/dl in serum creatinine level was associated with a 1.761-fold risk of mortality. Finally, the Kaplan-Meier analysis also revealed patients with acute kidney injury suffered lower cumulative survival than patients without acute kidney injury (Fig. 1, log-rank test, Chi-square = 5.836, P = 0.016).

## Discussion

The overall mortality rate in our charcoal burning suicide population was 3.2%. This favorable mortality figure was comparable with data from other poison centers. One previous study of charcoal burning suicide reported that their mortality rate was 4.1%^13^, and another study of CO poisoning reported 2.6%^10^. Furthermore, there was only 5% of in-hospital mortality even in patients with moderate to severe CO poisoning^11^. Additionally, our recent study of Taiwanese with CO poisoning reported a mortality rate of 7.3%^12^.

The analysis indicates that acute kidney injury was most strongly associated with a higher risk of mortality. In a Cox regression model, it was disclosed that serum creatinine level (P = 0.019) was a significant risk factor for mortality. Kaplan-Meier analysis also revealed patients with acute kidney injury suffered lower cumulative survival than patients without injury (P = 0.016). There were occasional case reports^14^,^15^,^16^ of acute kidney injury and rhabdomyolysis after CO poisoning. The affinity of carbon monoxide to hemoglobin was about 200 times greater than oxygen^17^, therefore CO poisoning would lead to hypoxia damage to human body. The CO mainly affects the central nervous system and the myocardium; acute kidney injury might occur due to acute rhabdomyolysis and hypoxia.

Interestingly, CO is also an endogenously produced gas resulting from the degradation of heme by heme oxygense or from fatty acid oxidation^18^. Recent researches using CO inhalation therapy and carbon monoxide releasing molecules have demonstrated that very small increase in CO could be beneficial to the kidney in several forms of acute renal injury by limiting oxidative injury, decreasing cell apoptosis, and promoting cell survival^18^.

In a Cox regression model, it was also disclosed that heart rate (P = 0.022) was a significant risk factor for mortality. Each increment of 1 beat per minute in heart rate was associated with a 1.117-fold risk of mortality. Since our body suffered hypoxic stress in CO poisoning due to high affinity of CO to hemoglobin^17^, and the systemic hypoxia could induce rapid heart rate so as to compensate for the stress.

The majorities of the charcoal burning suicide patients were young to middle age (36.8 ± 11.7 years), male (66.7%), whereas 46.0% was single, 11.9% were divorced, 11.9% living alone, 42.1% senior high school educated, but only 31.7% were jobless. In previous studies, people committed charcoal burning suicide were also mostly young to middle age^19^, male^2^,^13^,^20^,^21^,^22^,^23^, had job^13^,^24^, educated^21^,^22^, single or divorced^13^,^21^,^22^,^24^, but not living alone^25^. It was reported that the men-to-women ratio seemed to be greater in Taiwan than in Hong Kong^26^, and people committed charcoal burning suicide tended to have non-manual jobs^21^,^22^. Notably, a total of 21.4% of the patients had previous suicide history, 49.2% had depressive disorder, 39.7% had adjustment disorder, and 11.1% had substance abuse disorder. Other groups had also observed a high incidence of mood disorder in their studies^13^,^20^,^25^. Furthermore, the percentage of previous suicide history was reported as 33% in a study^25^, and 38.4% in another^13^. Overall, the baseline demographic characteristics of charcoal burning suicide in our study were similar to previous reports.

The carboxyhemoglobin level detected in hospital was greatly affected by the timing of hospital arrival because different patients had different time elapsed between termination of CO exposure to hospital arrival. The rescue intervention of oxygen supply before arriving to hospital might be another interference^27^. In the present study, the level of carboxyhemoglobin was not related to the mortality of charcoal burning suicide patients, and our finding was similar to a retrospective analysis of 1505 patients^10^. Also, there was no statistical difference between patients with and without acute kidney injury in term of carboxyhemoglobin level (P > 0.05). One study analyzed 1407 CO poisoning patients from 1978 to 2005 reported that compared to those survived, the carboxyhemoglobin level was significantly higher in 37 patients who died in 30 days. However, due to limitation of that study, it was suggested that the clinical correlation between carboxyhemoglobin level and medical conditions was not sure^27^. Furthermore, a 10-year-period study that divided 476 patients into 3 groups based on carboxyhemoglobin levels reported that no correlation was found between carboxyhemoglobin levels and vital signs^28^. One study also noted that the initial carboxyhemoglobin level was unrelated to subsequent cognitive sequel^29^. Nevertheless, the level of carboxyhemoglobin still has its clinical role. In order to rapidly estimate carboxyhemoglobin level, venous carboxyhemoglobin level could also be used due to it has high accuracy to predict arterial carboxyhemoglobin level^30^, and the pulse CO oximeter could be another choice for fast rescue management^31^.

In the present study, 52 cases (41.3%) received hyperbaric oxygen therapy. A Cox regression analysis revealed that hyperbaric oxygen therapy was not a significant factor that associated with good outcome. Previous papers mentioned the correlation between hyperbaric oxygen therapy and mortality rate were variable. Applying hyperbaric oxygen therapy was considered to use oxygen to compete the binding sites of hemoglobin, and to shorten the half-life of carboxyhemoglobin into 15–30 minutes^17^. Hyperbaric oxygen therapy seemed to have the potential to reduce hypoxic effects, and therefore oxygen-consuming organs were protected. One study reported hyperbaric oxygen therapy was better than normobaric oxygen therapy in reducing risk of cognitive squeal^32^. Another cell-based study reported hyperbaric oxygen had better protective effect over rat astrocytes^33^. Our data revealed that patients with acute kidney injury received less hyperbaric oxygen therapy than patients without acute kidney injury (26.5% versus 50.6%, P = 0.007). The reason was unclear. One possible explanation was since there were more incidences of acute respiratory failure in patients with acute kidney injury than without injury (49.0% versus 22.1%, P = 0.002); it was possible that these patients could not receive hyperbaric oxygen therapy simply because of intubation. Notably, a Cochrane systemic review of 6 studies reported that the existing data did not establish whether the administration of hyperbaric oxygen therapy to patients with carbon monoxide poisoning could reduce the incidence of adverse neurologic outcomes^34^.

## Conclusions and Limitations

In summary, the overall mortality rate in our charcoal burning suicide population was 3.2%. Furthermore, the analysis indicates that acute kidney injury was most strongly associated with a higher risk of mortality. Nevertheless, our data was limited by retrospective nature of the study, small sample size, single-center study, difficulty in obtaining the initial ambient carbon monoxide concentrations or carboxyhemoglobin levels at the scene, lack of measurement of serum inflammation markers, lack of psychoanalytical data, and finally lack of standard indications for hyperbaric oxygen therapy. Notably, the primary and most obvious shortcoming of most single-center studies is their potentially limited external validity. Results from a single clinical hospital are not necessarily generalizable to a broader population, and this may be particularly true in critically ill patients such as charcoal burning suicide. Further studies are warranted.

## Methods

### Ethics

The present retrospective observational study complied with the guidelines of the Declaration of Helsinki, and was approved by the Medical Ethics Committee of Chang Gung Memorial Hospital, a tertiary referral center (with 24-hour hyperbaric oxygen service) located in the northern part of Taiwan. Since this study involved a retrospective review of existing data, Institutional Review Board approval was obtained without specific informed consent from the patients. However, informed consent was obtained from all patients at their initial admission for risk of acute CO poisoning and all treatments. Additionally, all individual information was securely protected by delinking identifying information from main data set and was only available to investigators. Furthermore, all of the data were analyzed anonymously. The Institutional Review Board of the Chang Gung Memorial Hospital had specifically waived the need for consent. Finally, all primary data were collected according to the strengthening the reporting of observational studies in epidemiology guidelines. The studying method was used and based on previous studies^12^,^35^,^36^.

### Patients

We enrolled all patients with the diagnosis of acute CO poisoning with the intension of charcoal burning suicide at Chang Gung Memorial hospital from 2002 to 2013. Clinical history, clinical manifestations, physical examinations, and blood carboxyhemoglobin test were examined for the diagnosis of CO poisoning. Social demographic information such as gender, underlying diseases, personal habits, marriage status, educational status, working condition, living condition, and previous psychiatric diseases was examined. Medical data of enrolled patients including Glasgow coma scale, vital signs, clinical manifestations, radiographic image for detecting globus pallidus necrosis, and laboratory examinations such as blood test, carboxyhemoglobin test, electrolytes and arterial blood gas were collected. Moreover, the detoxification treatment, hospitalization duration, and mortality data were recorded.

### Inclusion and exclusion criteria

All patients committed charcoal burning suicide with the diagnosis of acute CO poisoning^37^ and sent to the emergency room of Chang Gung Memorial Hospital from 2002 to 2013 were included into the present study. We excluded CO poisoning patients with suicide intention by using other methods (i.e. wasting gas of cars, burning other materials) instead of charcoal burning.

### Detoxification protocol

Treatments included administering a high concentration of oxygen therapy via a non-rebreather mask or providing hyperbaric oxygen therapy. Similar to other international Poison Centers^38^, there was no standard indication for such hyperbaric oxygen treatment.

### Hyperbaric oxygen therapy

All patients breathed 100% oxygen via a facial mask at elevated pressure of 2.5 atmospheric absolute for 90–95 minutes daily. The absolute contraindication for hyperbaric oxygen therapy was an untreated pneumothorax. On the other hand, the relative contraindications included asthma, chronic obstructive pulmonary disease, history of seizure, high fever, upper respiratory infections, viral infections, pregnancy, active cancerous condition, congenital spherocytosis, claustrophobia, diseases related to ears, nose, and eyes, and those patients who need intensive care.

### Definitions of clinical events

Fever was defined as a body temperature of above 38.3 °C. Acute respiratory failure implied that a patient needed intubation and mechanical ventilation. Acute myocardial injury was defined as troponin-I of more than 5 ng/mL or abnormal electrocardiogram. Acute hepatic injury was defined as elevation of alanine aminotransferase level greater than 2 times of upper normal limit (i.e., >68 U/L, normal: 0–34 U/L) or total bilirubin levels of >1.5 mg/dL. Acute rhabdomyolysis was defined as increase of myoglobin in the urine, and marked elevation of total creatinine kinase for more than 5 times of upper limit (i.e., >10000 U/L, normal: 200 U/L)^39^. Shock was defined as a condition with an abnormality of the circulatory system that results in inadequate organ perfusion and tissue oxygenation. Globus pallidus necrosis was defined as bilateral and symmetric lesions in globus pallidus using brain radiographic studies such as magnetic resonance imaging^40^ or computed tomography^41^.

### Statistical analysis

All data were tested for normality of distribution and equality of standard deviation prior to analysis. Continuous variables were expressed as the means ± standard deviations for the number of observations. In the meantime, categorical variable were expressed as number (percentages). For comparison between two groups, the Student’s t test for quantitative variables and Chi-square or Fisher’s exact tests for categorical variables were used. The Kaplan-Meier method was applied for mortality comparison and significance was tested using a log-rank test. A univariate Cox regression analysis was performed to compare the frequency of possible risk factors associated with mortality. To control for confounding factors, a multivariate Cox regression analysis (stepwise backward approach) was performed with the factors that were significant in univariate models (P < 0.05) and met the assumptions of a proportional hazard model. We considered P < 0.05 as statistical significance. All statistical analyses were performed using IBM SPSS Statistics Version 20 (IBM Corporation, Armonk, NY, USA).

## Additional Information

**How to cite this article**: Chen, Y.-C. *et al*. Acute Kidney Injury Predicts Mortality after Charcoal Burning Suicide. *Sci. Rep.* **6**, 29656; doi: 10.1038/srep29656 (2016).

## Figures and Tables

**Table 1:** Baseline characteristics of patients with charcoal burning suicide, stratified according to status of renal damage as acute kidney injury (creatinine 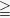
1.2 mg/dL) or non-acute kidney injury (creatinine < 1.2 mg/dL) group (N = 126).

**Variable**	**Acute kidney injury (N = 49)**	**Non-acute kidney injury (N = 77)**	**P value**
Age (year-old)	35.5 ± 12.2	37.6 ± 11.4	0.309
Male, n (%)	40 (81.6)	44 (57.1)	0.004**
Carboxyhemoglobin (g/dL)	20.7 ± 18.7	23.5 ± 17.9	0.408
Time elapsed between poisoning and hospital arrival (hour)	7.0 ± 11.0	7.9 ± 14.0	0.822
Hypertension, n (%)	2 (4.1)	9 (11.7)	0.140
Diabetes mellitus, n (%)	4 (8.2)	7 (9.1)	0.857
Chronic viral hepatitis, n (%)	3 (6.1)	5 (6.5)	0.934
Liver cirrhosis, n (%)	2 (4.1)	0 (0)	0.074
Chronic pulmonary disease, n (%)	3 (6.1)	4 (5.2)	0.825
Smoking habit, n (%)	29 (59.2)	32 (41.6)	0.019*
Alcohol consumption, n (%)	24 (49.0)	31 (40.3)	0.040*

Note: *P < 0.05, **P < 0.01, ***P < 0.001.

**Table 2:** Psychiatric comorbidities of patients with charcoal burning suicide, stratified according to status of renal damage as acute kidney injury (creatinine 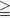
1.2 mg/dL) or non-acute kidney injury (creatinine < 1.2 mg/dL) group (N = 126).

**Variable**	**Acute kidney injury (N = 49)**	**Non-acute kidney injury (N = 77)**	**P value**
Marriage status			
Married, n (%)	9 (18.4)	33 (42.9)	0.009**
Single, n (%)	26 (53.1)	32 (41.6)	
Divorced, n (%)	10 (20.4)	5 (6.5)	
Widow, n (%)	0 (0)	3 (3.4)	
Education			
No, n (%)	1 (2.0)	1 (1.3)	0.527
Elementary school, n (%)	2 (4.1)	1 (1.3)	
Junior high school, n (%)	9 (18.4)	15 (19.5)	
Senior high school, n (%)	22 (44.9)	31 (40.3)	
University, n (%)	6 (12.2)	19 (24.7)	
Employed			
Jobless, n (%)	19 (38.8)	21 (27.3)	0.119
Manual labor, n (%)	3 (6.1)	19 (24.7)	
Service worker, n (%)	2 (4.1)	10 (13.0)	
Housewife, n (%)	1 (2.0)	4 (5.2)	
Army and police, n (%)	3 (6.1)	1 (1.3)	
Sale representative, n (%)	2 (4.1)	2 (2.6)	
Accountant, n (%)	1 (2.0)	3 (3.9)	
Retired, n (%)	1 (2.0)	3 (3.9)	
Administrative, n (%)	2 (4.1)	1 (1.3)	
Driver, n (%)	2 (4.1)	1 (1.3)	
Engineer, n (%)	0 (0)	3 (3.9)	
Student, n (%)	4 (8.2)	2 (2.6)	
Freelancer, n (%)	1 (2.0)	1 (1.3)	
Vender, n (%)	1 (2.0)	1 (1.3)	
Travel agent, n (%)	1 (2.0)	0 (0)	
Sex worker, n (%)	1 (2.0)	0 (0)	
Journalist, n (%)	1 (2.0)	0 (0)	
Medical professional, n (%)	0 (0)	1 (1.3)	
Living alone, n (%)	8 (16.3)	7 (9.1)	0.109
Previous suicide history, n (%)	12 (24.5)	15 (19.5)	0.348
Family psychiatric disorder, n (%)	2 (4.1)	3 (3.9)	0.959
Mood disorder			
Depressive disorder, n (%)	19 (38.8)	43 (55.8)	0.306
Bipolar disorder, n (%)	2 (4.1)	3 (3.9)	
Adjustment disorder, n (%)	22 (44.9)	28 (36.4)	0.297
Substance related psychiatric disorder, n (%)	8 (16.3)	6 (7.8)	0.171
Other psychiatric disorder			
Psychotic disorder, n (%)	1 (2.0)	4 (5.2)	0.862
Personality disorder, n (%)	1 (2.0)	1 (1.3)	
Anxiety, n (%)	1 (2.0)	1 (1.3)	
Dementia, n (%)	0 (0)	1 (1.3)	

Note: *P < 0.05, **P < 0.01, ***P < 0.001.

**Table 3:** Clinical manifestations of patients with charcoal burning suicide, stratified according to status of renal damage as acute kidney injury (creatinine 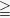
1.2 mg/dL) or non-acute kidney injury (creatinine < 1.2 mg/dL) group (N = 126).

**Variable**	**Acute kidney injury (N = 49)**	**Non-acute kidney injury (N = 77)**	**P value**
Glasgow Coma Scale			
Severe injury (3–8), n (%)	28 (57.1)	29 (37.7)	0.135
Moderate injury (9–12), n (%)	4 (8.2)	11 (14.3)	
Mild injury (13–15), n (%)	17 (34.7)	35 (45.5)	
Fever, n (%)	20 (40.8)	15 (19.5)	0.009**
Acute respiratory failure, n (%)	24 (49.0)	17 (22.1)	0.002**
Acute myocardial injury, n (%)	21 (42.9)	20 (26.0)	0.049*
Acute hepatic injury, n (%)	27 (55.1)	17 (22.1)	<0.001***
Acute rhabdomyolysis, n (%)	24 (49.0)	24 (31.2)	0.045*
Acute gastrointestinal upset, n (%)	6 (12.2)	4 (5.2)	0.154
Stroke, n (%)	5 (10.2)	3 (3.9)	0.157
Shock, n (%)	5 (10.2)	3 (3.9)	0.157
Out-of-hospital cardiac arrest, n (%)	3 (6.1)	0 (0)	0.028*
Burn injury (%)	0.9 ± 2.5	0.3 ± 1.5	0.112
Neuropsychological impairment, n (%)	21 (42.9)	26 (33.8)	0.538

Note: *P < 0.05, **P < 0.01, ***P < 0.001.

**Table 4:** Laboratory analysis of patients with charcoal burning suicide, stratified according to status of renal damage as acute kidney injury (creatinine 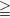 1.2 mg/dL) or non-acute kidney injury (creatinine < 1.2 mg/dL) group (N = 126).

**Variable**	**Acute kidney injury (N = 49)**	**Non-acute kidney injury (N = 77)**	**P value**
Vital signs			
Systolic blood pressure (mmHg)	116.4 ± 32.8	121.9 ± 26.8	0.308
Diastolic blood pressure (mmHg)	69.2 ± 22.3	73.5 ± 18.6	0.257
Heart rate (/minute)	102.2 ± 27.3	92.0 ± 23.3	0.03*
White blood count (/mm^3^)	19847.1 ± 9180.8	15112.0 ± 12834.5	0.027*
Polymorphs (%)	82.6 ± 12.9	81.6 ± 10.9	0.620
C reactive protein (mg/L)	65.7 ± 82.0	57.8 ± 90.6	0.778
Hemoglobin (g/dL)	15.3 ± 2.3	14.2 ± 2.2	0.008**
Hematocrit (%)	44.1 ± 6.0	41.2 ± 5.7	0.009**
Platelet count (10^3^/mm^3^)	232.7 ± 80.8	230.9 ± 61.2	0.888
Blood urea nitrogen (mg/dL)	31.2 ± 33.7	11.3 ± 2.8	<0.001***
Creatinine (mg/dL)	2.7 ± 2.4	0.9 ± 0.24	<0.001***
Creatine kinase (MB) (ng/mL)	651.2 ± 2187.5	29.5 ± 87.61	0.052
Creatine kinase (total) (U/L)	63010.5 ± 11317.0	8664.7 ± 1291.7	0.011*
Myoglobin (ng/mL)	24012.9 ± 59481.9	1305.3 ± 5343.1	0.022*
Urine myoglobin (ng/mL)	1125308.1 ± 1402720.7	206308.1 ± 459525.7	0.208
Troponin I (ng/mL)	5.4 ± 7.5	1.8 ± 4.9	0.003**
Aspartate aminotransferase (U/L)	404.5 ± 966.5	72.2 ± 93.1	0.005**
Alanine aminotransferase (U/L)	200.4 ± 400.5	49.0 ± 44.4	0.016*
Alkaline phosphatase (U/L)	67.3 ± 23.1	55.8 ± 37.7	0.365
Total bilirubin (mg/dL)	1.2 ± 1.0	0.5 ± 0.2	0.030*
Albumin (g/dL)	3.3 ± 1.1	3.5 ± 0.6	0.536
Calcium (mg/dL)	7.9 ± 1.1	8.2 ± 0.6	0.278
Phosphate (mg/dL)	3.9 ± 1.9	2.8 ± 0.9	0.032*
Sodium (mmol/L)	140.7 ± 4.8	140.7 ± 2.7	0.983
Potassium (mmol/L)	6.8 ± 17.1	4.5 ± 5.1	0.311
Arterial blood gas			
pH	7.3 ± 0.1	7.4 ± 0.6	< 0.001***
PCO_2_ (mmHg)	35.1 ± 8.8	34.9 ± 5.3	0.878
PO_2_ (mmHg)	241.0 ± 153.6	227.2 ± 137.5	0.610
HCO_3_ (mmol/L)	18.6 ± 5.1	21.8 ± 3.5	<0.001**
SaO_2_ (%)	95.6 ± 9.9	95.7 ± 11.7	0.967
Urine benzodiazepine, n (%)	8 (16.3%)	16 (20.8%)	0.371
Urine ethanol, n (%)	1 (2.0%)	7 (9.1%)	0.125
Urine amphetamine, n (%)	3 (6.1%)	2 (2.6%)	0.143
Urine morphine, n (%)	2 (4.1%)	0 (0.0%)	0.047*
Globus pallidus necrosis on imaging, n (%)	20 (40.8%)	20 (26.0%)	0.218

Note: *P < 0.05, **P < 0.01, ***P < 0.001.

**Table 5:** Detoxification protocol and outcome for patient with charcoal burning suicide, stratified according to status of renal damage as acute kidney injury (creatinine 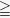
1.2 mg/dL) or non-acute kidney injury (creatinine < 1.2 mg/dL) group (N = 126).

**Variable**	**Acute kidney injury (N = 49)**	**Non-acute kidney injury (N = 77)**	**P value**
Hyperbaric oxygen therapy, n (%)	13 (26.5)	39 (50.6)	0.007**
Duration of hospitalization (days)	16.9 ± 18.3	10.7 ± 10.9	0.002**
Mortality, n (%)	4 (8.2)	0 (0)	0.011*

Note: *P < 0.05, **P < 0.01, ***P < 0.001.

**Table 6:** A Cox regression model for analysis of mortality (N = 126).

	**Uni-variable analysis**	**P value**	**Multi-variable analysis**	**P value**
	**Odds ratio (95% confidence interval)**		**Odds ratio (95% confidence interval)**	
Creatinine (each increase of 1 mg/dL)	1.474 (1.185–1.834)	<0.001***	1.761 (1.097–2.828)	0.019*
Heart rate (each increase of 1 beat per minute)	1.063 (1.022–1.106)	0.002**	1.117 (1.016–1.228)	0.022*
Bicarbonate (each decrease of 1 mmol/L)	1.235 (1.027–1.484)	0.025*	1.224 (0.810–1.852)	0.337

*P < 0.05, **P < 0.01, ***P < 0.001.
